# Case Report: Severe Edema and Marked Weight Gain Induced by Marginal Thiamine Deficiency in a Patient With Alcohol Dependency and Type 2 Diabetes Mellitus

**DOI:** 10.3389/fnut.2021.675992

**Published:** 2021-12-15

**Authors:** Hitomi Tanaka, Takatoshi Anno, Haruka Takenouchi, Hideyuki Iwamoto, Hideaki Kaneto, Niro Okimoto, Koichi Tomoda

**Affiliations:** ^1^Department of General Internal Medicine, Kawasaki Medical School, Okayama, Japan; ^2^Department of Diabetes, Endocrinology and Metabolism, Kawasaki Medical School, Kurashiki, Japan

**Keywords:** thiamine deficiency, alcohol dependency, whole body edema, weight gain, type 2 diabetes mellitus

## Abstract

**Background:** Patients with alcohol use disorder (AUD) may develop peripheral edema due to alcohol-related liver, renal, or heart disease. Thiamine deficiency is reported to occur in AUD and type 2 diabetes mellitus (T2DM). Thiamine deficiency may also cause peripheral edema. Thiamine is essential for optimal glucose metabolism through its role as an essential co-factor for key enzymes in intermediary metabolism. Since glucose metabolism worsens under diabetic conditions, it seems that a relative shortage of thiamine may occur more easily in patients with diabetes mellitus.

**Case Presentation:** A 59-year-old Japanese man was admitted to the hospital with severe peripheral edema. His background history included alcohol liver disease (ALD), chronic renal failure (CRF), and T2DM. His body mass index (BMI) at admission was 37.7 kg/m^2^ and this represented a 30 kg increase in body weight over 2 months. Laboratory investigations showed anemia, liver and renal injury, hyperglycemia, and marginal hypothyroidism. The plasma thiamine diphosphate concentration was 20 ng/mL (reference range: 24–66 ng/mL). Diet therapy of 1,600 kcal/day and intravenous fursultiamine hydrochloride therapy (50 mg/once a day, seven days) was commenced in combination with intravenous diuretics. After one week, the plasma thiamine concentration was 853 ng/mL, and the patient's body weight had reduced by 18 kg.

**Conclusions:** Patients with T2DM and AUD may develop severe peripheral edema in the context of marginal thiamine deficiency. Fursultiamine hydrochloride (50 mg/once a day, seven days) restored normal plasma thiamine concentrations and may have contributed to the rapid resolution of severe peripheral edema in this case. Empirical treatment with thiamine should be considered in patients with severe peripheral edema in the context of AUD and T2DM.

## Background

Alcohol dependency may cause peripheral edema, ascites, and heart failure. There are a variety of causes for fluid retention in patients with alcohol dependency. For example, ascites may be caused by severe alcohol-related liver injury or cirrhosis, which is often complicated with alcohol dependency (1). Thiamine (vitamin B1) deficiency may also cause peripheral edema and severe thiamine deficiency may progress to cardiac failure-related edema (2).

Furthermore, thiamine is essential for optimal glucose metabolism through its role as an essential co-factor for pyruvate dehydrogenase (PDH) and alpha-ketoglutarate dehydrogenase (AKGDH). PDH catalyzes the conversion of pyruvate to acetyl CoA and therefore represents the bridge between glycolysis and the tricarboxylic acid (TCA) cycle (3, 4). There are few reports of thiamine and thiamine-dependent enzyme status in clinical diabetes mellitus. It has been reported that thiamine-dependent enzyme transketolase (TK) activity is lower in patients with diabetes and the concentration of vitamin B1 is lower in patients with diabetes (5–7). In addition, it has been reported that glucose tolerance at least in part, is influenced by dietary thiamine intake (7, 8). Furthermore, glucose metabolism is reported to deteriorate in patients with type 2 diabetes mellitus (T2DM) in the context of thiamine deficiency (7).

Here we describe a patient with alcohol dependency, T2DM, and marginal thiamine deficiency who suffered from severe edema in the whole body and marked weight gain. His fluid retention was very severe and his body weight was increased by over 30 kg during the preceding 2 months. His severe peripheral edema was resolved after 2 weeks following treatment of thiamine deficiency.

## Case Presentation

A 59-year-old Japanese man was admitted to the hospital with symptoms of diffuse peripheral edema. He reported general fatigue and severe edema that had evolved over the preceding 2 months. Moreover, his body weight was increased by over 30 kg (body weight on admission = 101.5 kg). At that time, his height and body mass index (BMI) were 164.0 cm and 37.7 kg/m^2^, respectively. His background history included alcohol dependency for 30 years, previous alcohol-related pancreatitis (at the age of 25), hypertension for 10 years, a recent diagnosis of Parkinson's disease (at 53), and alcohol-related liver disease (at 55). His medications included nifedipine (20 mg/once a day) for hypertension and rotigotine (18 mg/once a day), levodopa/carbidopa (100 mg/three times a day), and tandospirone (10 mg/three times a day) for Parkinson's disease. In addition, he was taking three tablets/day of excelase combination tablets, branched-chain amino acid (4.5 g/three times a day), ursodeoxycholic acid (100 mg/three times a day), furosemide (20 mg/once a day), and spironolactone (25 g/once a day) for the treatment of pancreatitis, alcoholic liver injury, and edema. His father had Parkinson's disease and his mother had diabetes mellitus. He was a smoker (pack-years = 2 packs/day × 43 years). His daily intake of alcohol was 540 mL of Japanese liquor called Sake or Shochu. As his daily intake of alcohol was increased, his body weight was increased. His vital signs were: temperature, 37.1°C; blood pressure, 160/90 mmHg; heart rate, 95 bpm; oxygen saturation, 97%. Table 1 shows the laboratory data on admission. Infection markers were mildly elevated: white blood cell, 7,400/μL (reference interval: 3,300–8,600/μL); C-reactive protein, 2.79 mg/dL (<0.14 mg/dL). His albumin was decreased [3.0 g/dL (4.1–5.1 mg/dL)]. Renal and liver dysfunction was observed: creatinine, 1.32 mg/dL (0.65–1.07 mg/dL); blood urea nitrogen (BUN), 17 mg/dL (8–20 mg/dL); aspartate aminotransferase (AST), 52 U/L (13–30 U/L); alanine transaminase (ALT), 25 U/L (10–42 U/L); alkaline phosphatase (ALP), 300 U/L (106–322 U/L); γ-glutamyl transpeptidase (γ-GTP), 190 U/L (13–64 U/L); lactate dehydrogenase (LDH), 195 U/L (124–222 U/L). Marginal iron deficiency anemia was observed. Nutrient deficiency-associated data showed marginal thiamine deficiency (Table 1). He had liver and renal injuries. Although he did not take any antidiabetic drug on admission, he had T2DM: plasma glucose, 132 mg/dL; hemoglobin A1c, 7.1 % (4.9–6.0 %). In addition, he had marginal hypothyroidism: thyroid-stimulating hormone (TSH), 8,228 μIU/mL (0.400–6.000 μIU/mL); free tri-iodothyronine (FT3), 3.03 pg/mL (2.50–4.20 pg/mL); free thyroxine (FT4) 0.69 ng/dL (0.80–1.60 ng/dL). Since his hypothyroidism was subclinical hypothyroidism, we observed it without levothyroxine therapy. The brain natriuretic peptide (BNP) concentration was mildly elevated [35.5 pg/mL (0.0–18.4 pg/mL)].

**Table 1:** Laboratory data on admission in this patient.

**Variable**	**Result**	**Reference range**
**Peripheral blood**		
White blood cells (/μL)	7,400	3,300–8,600
Red blood cells (× 10^4^/μL)	357	435–555
Hemoglobin (g/dL)	9.9	13.7–16.8
Reticulocyte (%)	2.1	0.5–2.0
MCV (fL)	89.6	83.6–98.2
MCH (pg)	27.7	27.5–33.2
MCHC (g/dL)	30.9	31.7–35.3
Platelets (× 10^4^/μL)	16.8	15.8–34.8
**Blood biochemistry**		
Total protein (g/dL)	8.1	6.6–8.1
Albumin (g/dL)	3.0	4.1–5.1
Globulin (g/dL)	5.1	2.2–3.4
Total bilirubin (mg/dL)	0.6	0.4–1.5
AST (U/L)	52	13–30
ALT (U/L)	25	10–42
LDH (U/L)	195	124–222
ALP (U/L)	300	106–322
γ-GTP (U/L)	190	13–64
BUN (mg/dL)	17	8–20
Creatinine (mg/dL)	1.32	0.65–1.07
Cholinesterase (U/L)	169	240–486
Uric acid (mg/dL)	10.0	3.7–7.8
Creatine kinase (U/L)	146	59–248
Amylase (U/L)	75	44–132
Pancreatic amylase (U/L)	20	19–53
CRP (mg/dL)	2.79	<0.14
BNP (pg/mL)	35.5	0.0–18.4
**Electrolytes**		
Sodium (mmol/L)	139	138–145
Potassium (mmol/L)	3.8	3.6–4.8
Chloride (mmol/L)	105	101–108
IP (mg/dL)	2.7	2.7–4.6
Calcium (mg/dL)	8.4	8.8–10.1
Magnesium (mg/dL)	1.6	1.9–2.6
**Anemia marker**		
Iron (μg/dL)	31	40–188
TIBC (μg/dL)	410	248–395
UIBC (μg/dL)	379	70–282
Tf % (%)	7.9	
Ferritin (ng/mL)	19	10–240
Vitamin B12 (pg/mL)	1211	233–914
**Vitamin**		
Vitamin B1 (ng/mL)	20	24–66
Vitamin B2 (ng/mL)	79.9	66.1–111.4
**Metabolism and Endocrinology marker**		
Plasma glucose (mg/dL)	132	
Hemoglobin A1c (%)	7.1	4.9–6.0
Total cholesterol (mg/dL)	161	142–248
LDL cholesterol (mg/dL)	101	65–139
HDL cholesterol (mg/dL)	36	40–90
Triglyceride (mg/dL)	90	40–149
TSH (μIU/mL)	8.228	0.400–6.000
FT3 (pg/mL)	3.03	2.50–4.20
FT4 (ng/dL)	0.69	0.80–1.60

*MCV, mean corpuscular volume; MCH, mean corpuscular hemoglobin; MCHC, mean corpuscular hemoglobin concentration; AST, aspartate aminotransferase; ALT, alanine aminotransferase; LDH, lactate dehydrogenase; ALP, alkaline phosphatase; γ-GTP, γ-glutamyl transpeptidase; BUN, blood urea nitrogen; CRP, C-reactive protein; BNP, brain natriuretic peptide; IP, Inorganic Phosphorus; TIBC, total iron binding capacity; UIBC, unsaturated iron binding capacity; Tf%, transferrin % saturation; LDL, Low-density lipoproteins; HDL, High-density lipoprotein; TSH, thyroid stimulating hormone; FT3, free triiodothyronine; FT4, free thyroxine*.

**Figure 1:**
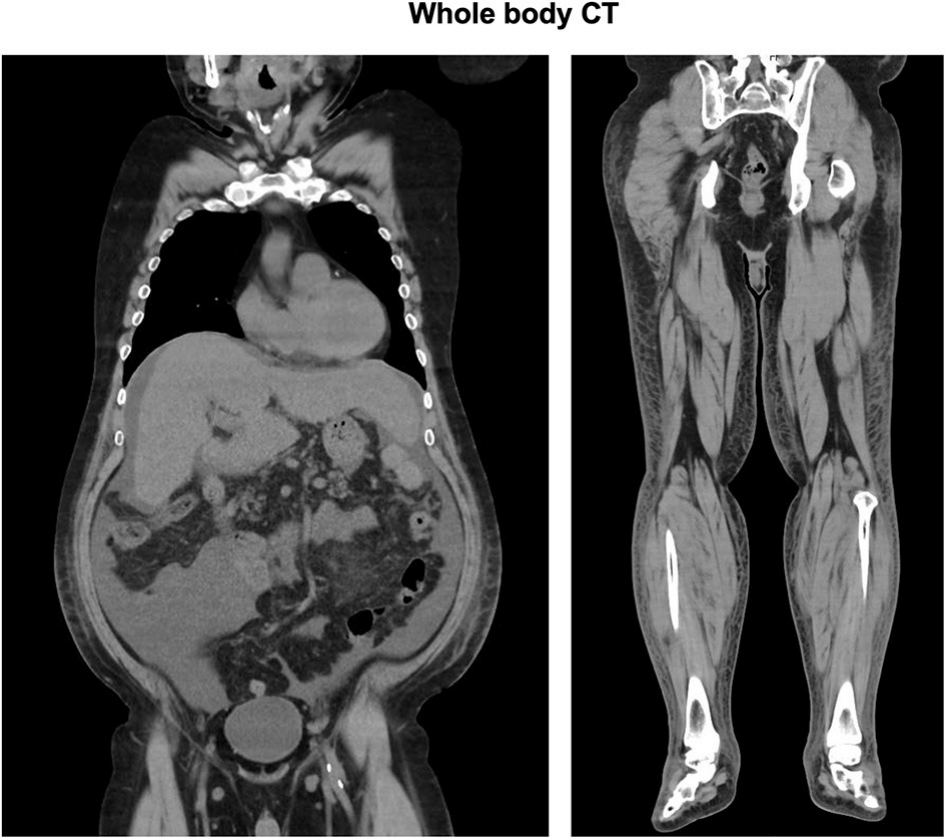
Total body CT: Chest CT showed a marginal pleural effusion and cardiomegaly. Abdominal CT revealed early liver cirrhosis together with fatty change, surface nodularity, and a small amount of ascites. Limb CT revealed severe subcutaneous edema mainly in the lower limb.

**Figure 2:**
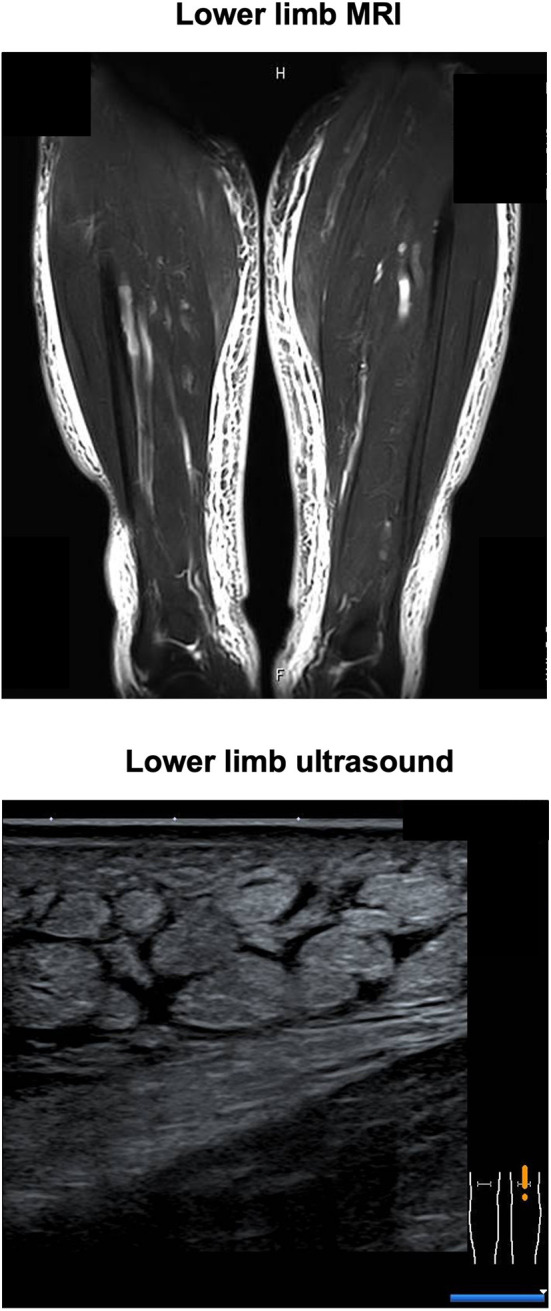
Magnetic resonance imaging (MRI) and ultrasonography showed lower limb edema.

His CT showed a marginal pleural effusion and cardiomegaly. His abdominal CT revealed early liver cirrhosis together with fatty change, surface nodularity, and a small amount of ascites. In addition, limb CT revealed severe subcutaneous edema mainly in the lower limb (Figure 1). It seemed that his effusion and edema were mainly due to lower limb edema and partially due to pleural effusion, cardiomegaly, and ascites on MRI and ultrasonography (Figure 2). The electrocardiogram was normal except for the left axis deviation. The echocardiography demonstrated no remarkable change compared with that examined four years before, with a left ventricular ejection fraction of 61–63 %. Marked right atrial and ventricular dilation was found, although noted to be unchanged relative to the previous echo examination four years previously. His liver cirrhosis and heart failure were not very severe compared with his leg edema and weight gain. Based on such findings, we think it is possible that severe edema in the whole body and significant weight gain were, at least in part, induced by thiamine deficiency itself, although he had various causes of fluid retention due to alcohol dependency.

**Figure 3:**
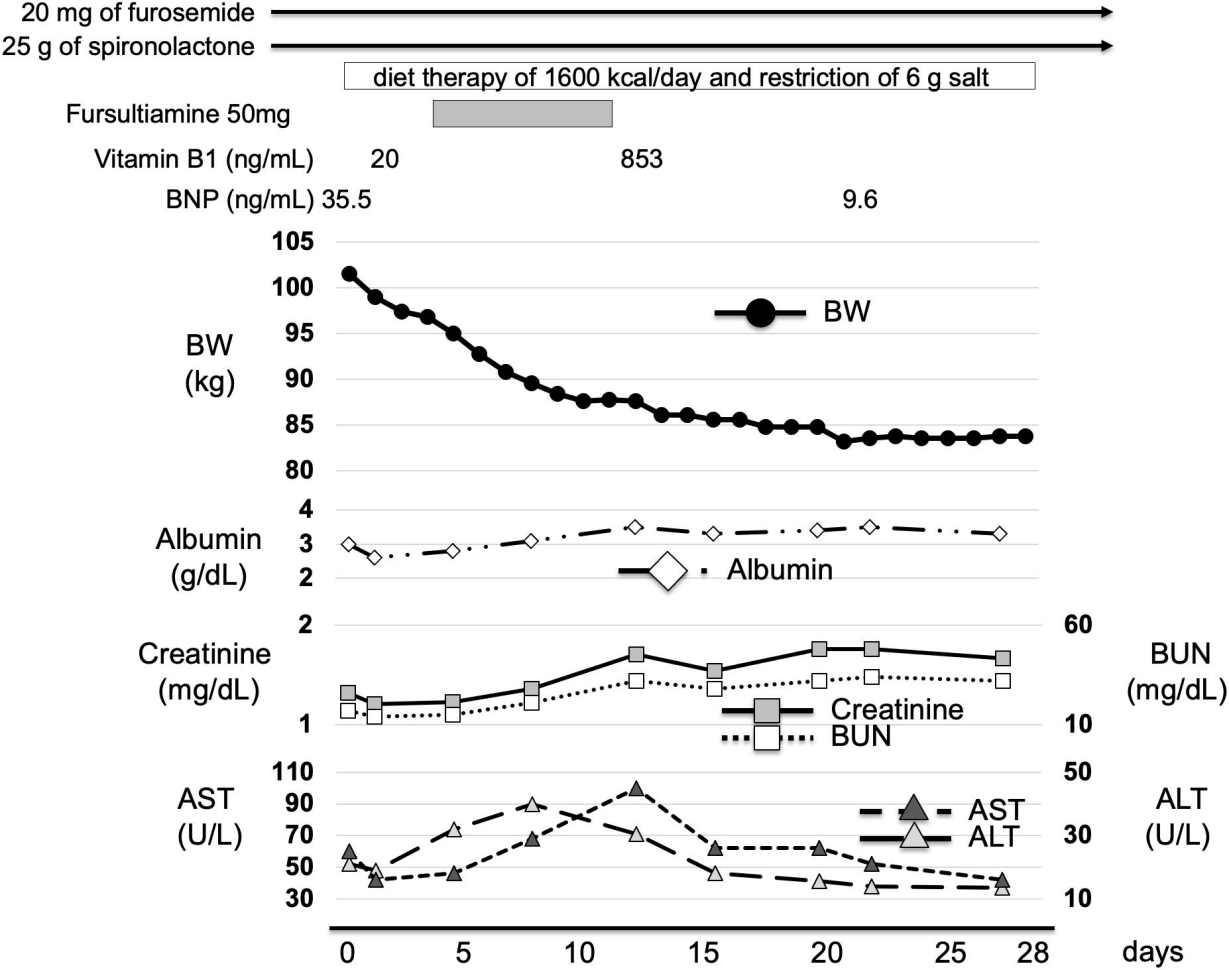
Time course of clinical parameters in this patient. We continued 20 mg/day of furosemide and 25 g/day of spironolactone treatment for edema. He was treated with diet therapy of 1,600 kcal/day and a restriction of 6 g of salt after admission. In addition, we started 50 mg/day of fursultiamine hydrochloride. Plasma vitamin B1 concentration was increased from 20 to 853 ng/mL. His body weight was decreased by 18 kg only after two weeks and it was 83.2 kg at discharge. BW, body weight; BUN, blood urea nitrogen; AST, aspartate aminotransferase; ALT, alanine aminotransferase.

Caloric restriction diet was commenced immediately after admission. Nifedipine, furosemide, and spironolactone medications were also continued. In addition, intravenous fursultiamine hydrochloride therapy (50 mg/once a day) was also commenced four days after admission. According to the dietary history from the patient, he consumed a total of 1,300 kcal/day of meals, which was mainly carbohydrate, and over 500 kcal/day of alcohol every day. First, we treated him with diet therapy of 1,600 kcal/day (about 27 kcal/ideal body weight kg) and a restriction of 6 g salt for T2DM and hypertension. In Japan, it is recommended to have 25–30 kcal/ideal body weight (kg) for patients with diabetes mellitus. We continued 20 mg/day of nifedipine for hypertension and diet therapy only and no medication for T2DM. We continued furosemide (20 mg/once a day) and spironolactone (25 g/once a day) treatment for edema. In addition, since we considered that the severe edema in the whole body and significant weight gain were, at least in part, caused by thiamine deficiency, we started fursultiamine hydrochloride (50 mg/once a day) for seven days. The plasma vitamin B1 concentration was increased to 853 ng/mL (reference interval: 24–66 ng/mL) (Liquid chromatography-tandem mass spectrometry, SRL Inc., Tokyo). Table 2 shows the laboratory data at a point when the vitamin B1 concentration was increased (12 days after admission). His body weight was decreased by 18 kg two weeks later and was 83.2 kg at discharge. His conditions of the heart, liver, and renal function did not substantially change during the period of exacerbation of his edema and weight gain. In addition, ALT, AST, creatinine, and BUN were rather increased mildly probably due to dehydration 12 days after admission when body weight was decreased (Tables 1, 2; Figure 3). Thereafter, his liver and renal function were gradually improved (Figure 3). These results suggest the possibility that the significant decline in body weight in this patient was, at least in part, attributed to the treatment of mild thiamine deficiency.

**Table 2:** Laboratory data on 12 days after admission in this patient.

**Variable**	**Result**	**Reference range**
**Peripheral blood**		
White blood cells (/μL)	6,850	3,300–8,600
Red blood cells (× 10^4^/μL)	392	435–555
Hemoglobin (g/dL)	10.8	13.7–16.8
MCV (fL)	87.2	83.6–98.2
MCH (pg)	27.6	27.5–33.2
MCHC (g/dL)	31.6	31.7–35.3
Platelets (× 10^4^/μL)	16.2	15.8–34.8
**Blood biochemistry**		
Total protein (g/dL)	9.1	6.6–8.1
Albumin (g/dL)	3.5	4.1–5.1
Globulin (g/dL)	5.6	2.2–3.4
Total bilirubin (mg/dL)	0.7	0.4–1.5
AST (U/L)	71	13–30
ALT (U/L)	45	10–42
LDH (U/L)	204	124–222
ALP (U/L)	328	106–322
γ-GTP (U/L)	145	13–64
BUN (mg/dL)	32	8–20
Creatinine (mg/dL)	1.71	0.65–1.07
Cholinesterase (U/L)	191	240–486
Uric acid (mg/dL)	10.8	3.7–7.8
Creatine kinase (U/L)	115	59–248
CRP (mg/dL)	1.08	<0.14
**Electrolytes**		
Sodium (mmol/L)	132	138–145
Potassium (mmol/L)	4.4	3.6–4.8
Chloride (mmol/L)	100	101–108
IP (mg/dL)	3.9	2.7–4.6
Calcium (mg/dL)	8.7	8.8–10.1
Magnesium (mg/dL)	1.0	1.9–2.6
**Vitamin**		
Vitamin B1 (ng/mL)	853	24–66
**Metabolism and Endocrinology marker**		
Plasma glucose (mg/dL)	103	
Total cholesterol (mg/dL)	153	142–248

*MCV, mean corpuscular volume; MCH, mean corpuscular hemoglobin; MCHC, mean corpuscular hemoglobin concentration; AST, aspartate aminotransferase; ALT, alanine aminotransferase; LDH, lactate dehydrogenase; ALP, alkaline phosphatase; γ-GTP, γ-glutamyl transpeptidase; BUN, blood urea nitrogen; CRP, C-reactive protein; IP, Inorganic Phosphorus*.

## Discussion

Herein, we reported a case with severe edema in the whole body and significant weight gain induced by marginal thiamine deficiency complicated with alcohol dependency. In general, in patients with alcohol dependency, especially with liver cirrhosis, fluid retention such as ascites is often observed. There are several possible mechanisms for such fluid retention as follows: the overflow of fluid caused by excessive sodium and fluid retention in the kidney (9), some underfilling caused by the increase of sodium and fluid resorption together with the activation of the renin-angiotensin-aldosterone system due to decreased effective circulating blood volume (10), and substantial dilation of the peripheral artery caused by decreased effective circulating blood volume due to portal hypertension (11). Hypoalbuminemia and portal hypertension are also associated with fluid retention in patients with liver dysfunction. Diuretics such as furosemide and spironolactone are usually used in the treatment of fluid retention in this patient group (12). In this patient, based on such recommended treatment procedures, furosemide and spironolactone had been used to mitigate severe edema. Nonetheless, severe whole-body edema in the whole body and significant weight gain had developed in this patient over two months before presentation. Moreover, the patient reported that as his daily intake of alcohol was also increased, his body weight was increased. Although his Child-Pugh Score for Cirrhosis Mortality, was relatively elevated (seven points), this score did not change after the start of severe edema and weight gain. Therefore, it was not likely that his severe edema and weight gain were induced by the aggravation of liver function.

Thiamine deficiency is a medical condition of low concentrations of plasma thiamine (vitamin B1). In our hospital, the reference range of plasma thiamine is from 24 to 66 ng/ml. However, in general, thiamine deficiency is defined as <20 ng/mL, and low concentrations of thiamine are from 20 to 27 ng/mL. It is known that thiamine deficiency becomes one of the causes of various edema (13), brain cytotoxic edema and vasogenic edema (14, 15), and local edema in muscles (16). The most common edema associated with thiamine deficiency is caused by heart disorders, which is called wet beriberi. Beriberi is one of the clinical syndromes associated with thiamine deficiency. In addition, it is known that beriberi-associated edema is hemodynamically characterized by high output heart failure with low peripheral vascular resistance (17). It is recommended that we should treat the patients using fursultiamine hydrochloride if we suspect wet beriberi due to heart failure (18). However, his heart function did not change for four years on echocardiography and his BNP concentration was not elevated (35.5 pg/mL). It seemed that he did not suffer from severe heart failure. In addition, he was not complicated with Wernicke's encephalopathy. Therefore, we think it is possible that severe edema in the whole body and significant weight gain in this patient were, at least in part, induced by marginal thiamine deficiency itself. However, since edema can be induced by multiple causes such as mild renal impairment, liver failure, and hypothyroidism, we cannot exclude the possibility that some of these conditions contributed to the evolution of edema in this patient.

In addition, the long-term administration of the diuretic drug for heart failure may lead to thiamine deficiency through increased thiamine excretion (19). It is reported that thiamine supplementation may be effective for patients with heart failure (20), although still, there is not enough evidence. Fursultiamine hydrochloride is a nutritional supplement and vitamin B1 derivative. Since fursultiamine hydrochloride is used effectively inside the body, fursultiamine hydrochloride is administered as treatment or prevention of thiamine deficiency. Hence, fursultiamine hydrochloride may have contributed to the resolution of severe edema in this patient.

In patients with diabetes mellitus, glucose metabolism is aggravated to a greater or less extent. Therefore, it is likely that a larger amount of thiamine is needed under diabetic conditions and thereby relative shortage of thiamine is more easily brought about in patients with diabetes mellitus. We assume that this could explain, at least in part, the reason why marginal thiamine deficiency led to severe edema and marked weight gain in this patient. In addition, we assume that starting diet therapy in addition to stopping alcohol after admission enhanced the effects of fursultiamine hydrochloride therapy by improving glucose metabolism.

There is a limitation in this case report. First, the precise causative link between the lower thiamine concentrations and this patient's conditions remained unclear. We assume that a mildly lower value of thiamine is more pathogenic if thiamine transport is impaired due to some mutations whose pathogenicity is not obvious under normal conditions (21). Second, we cannot exclude the possibility that his severe edema and weight gain were brought about by mixed conditions with various effects of alcohol dependency such as liver cirrhosis, marginal heart failure and hypertension, renal dysfunction, mineral unbalance, hypoalbuminemia, and diabetes mellitus.

We should bear in mind that patients with marginal thiamine deficiency in the context of alcohol dependency may present with severe peripheral and central edema with associated significant weight gain. This may be more likely to occur in the context of co-existing underlying diabetic conditions, even in the absence of liver cirrhosis and/or heart failure. Caloric restriction in combination with diuretic and fursultiamine hydrochloride therapy has been proven very effective for such edema and weight gain in this patient with alcohol-dependency, diabetes mellitus, and thiamine deficiency. Empirical treatment with thiamine should be considered in patients with severe peripheral edema in the context of AUD and T2DM.

## Data Availability Statement

The original contributions presented in the study are included in the article/supplementary material, further inquiries can be directed to the corresponding author/s.

## Ethics Statement

Written informed consent was obtained from the individual(s) for the publication of any potentially identifiable images or data included in this article.

## Author Contributions

HTan and TA researched data and wrote the manuscript. HTak and HI researched data and contributed to the discussion. HK, NO, and KT reviewed the manuscript. All authors have read and approved the manuscript.

## Conflict of Interest

The authors declare that the research was conducted in the absence of any commercial or financial relationships that could be construed as a potential conflict of interest.

## Publisher's Note

All claims expressed in this article are solely those of the authors and do not necessarily represent those of their affiliated organizations, or those of the publisher, the editors and the reviewers. Any product that may be evaluated in this article, or claim that may be made by its manufacturer, is not guaranteed or endorsed by the publisher.

## References

[1] Kashani A Landaverde C Medici V Rossaro L. Fluid retention in cirrhosis: pathophysiology and management. QJM. (2008) 101:71–85. 10.1093/qjmed/hcm121 18184668

[2] Hirota Y Kane RL Abelmann WH. Cardiovascular effects of exercise in hamsters with experimental thiamine deficiency. Jpn Circ J. (1979) 43:99–106. 10.1253/jcj.43.99 221700

[3] Kamal M Abbasy AJ Muslemani AA Bener A. Effect of nicotinamide on newly diagnosed type 1 diabetic children. Acta Pharmacol Sin. (2006) 27:724–7. 10.1111/j.1745-7254.2006.00313.x 16723091

[4] Thornalley PJ Babaei-Jadidi R Al Ali H Rabbani N Antonysunil A Larkin J. High prevalence of low plasma thiamine concentration in diabetes linked to a marker of vascular disease. Diabetologia. (2007) 50:2164–70. 10.1007/s00125-007-0771-4 17676306 PMC1998885

[5] Saito N Kimura M Kuchiba A Itokawa Y. Blood thiamine levels in outpatients with diabetes mellitus. J Nutr Sci Vitaminol (Tokyo). (1987) 33:421–30. 10.3177/jnsv.33.421 3451944

[6] Havivi E Bar On H Reshef A Stein P Raz I. Vitamins and trace metals status in non insulin dependent diabetes mellitus. Int J Vitam Nutr Res. (1991) 61:328–33. 1806538

[7] Thornalley PJ. The potential role of thiamine (vitamin B1) in diabetic complications. Curr Diabetes Rev. (2005) 1:287–98. 10.2174/157339905774574383 18220605

[8] Bakker SJ Hoogeveen EK Nijpels G Kostense PJ Dekker JM Gans RO. The association of dietary fibres with glucose tolerance is partly explained by concomitant intake of thiamine: the Hoorn Study. Diabetologia. (1998) 41:1168–75. 10.1007/s001250051047 9794103

[9] Lieberman FL Ito S Reynolds TB. Effective plasma volume in cirrhosis with ascites. Evidence that a decreased value does not account for renal sodium retention, a sponteneous reduction in glomerular filtration rate (GFR), and a fall in GFR during drug-induced diuresis. J Clin Invest. (1969) 48:975–81. 10.1172/JCI106078 5771197 PMC322311

[10] Witte CL Witte MH Dumont AE. Lymph imbalance in the genesis and perpetuation of the ascites syndrome in hepatic cirrhosis. Gastroenterology. (1980) 78:1059–68. 10.1016/0016-5085(80)90793-3 7380179

[11] Schrier RW Arroyo V Bernardi M Epstein M Henriksen JH Rodés J. Peripheral arterial vasodilation hypothesis: a proposal for the initiation of renal sodium and water retention in cirrhosis. Hepatology. (1988) 8:1151–7. 10.1002/hep.1840080532 2971015

[12] Moore KP Aithal GP. Guidelines on the management of ascites in cirrhosis. Gut. (2006) 55:vi1–12. 10.1136/gut.2006.099580 16966752 PMC1860002

[13] Whitfield KC Bourassa MW Adamolekun B Bergeron G Bettendorff L Brown KH. Thiamine deficiency disorders: diagnosis, prevalence, and a roadmap for global control programs. Ann N Y Acad Sci. (2018) 1430:3–43. 10.1111/nyas.13919 30151974 PMC6392124

[14] Jung YC Chanraud S Sullivan EV. Neuroimaging of Wernicke's encephalopathy and Korsakoff's syndrome. Neuropsychol Rev. (2012) 22:170–80. 10.1007/s11065-012-9203-4 22577003 PMC4728174

[15] Chandrakumar A Bhardwaj A 't Jong GW. Review of thiamine deficiency disorders: Wernicke encephalopathy and Korsakoff psychosis. J Basic Clin Physiol Pharmacol. (2018) 30:153–62. 10.1515/jbcpp-2018-0075 30281514

[16] Murate K Mizutani Y Maeda T Nagao R Kikuchi K Shima S. Patient with thiamine deficiency exhibiting muscle edema suggested by MRI. Front Neurol. (2018) 9:1083. 10.3389/fneur.2018.01083 30619043 PMC6297209

[17] Attas M Hanley HG Stultz D Jones MR McAllister RG. Fulminant beriberi heart disease with lactic acidosis: presentation of a case with evaluation of left ventricular function and review of pathophysiologic mechanisms. Circulation. (1978) 58:566–72. 10.1161/01.CIR.58.3.566 679449

[18] Donnino MW Vega J Miller J Walsh M. Myths and misconceptions of Wernicke's encephalopathy: what every emergency physician should know. Ann Emerg Med. (2007) 50:715–21. 10.1016/j.annemergmed.2007.02.007 17681641

[19] Keith ME Walsh NA Darling PB Hanninen SA Thirugnanam S Leong-Poi H. B-vitamin deficiency in hospitalized patients with heart failure. J Am Diet Assoc. (2009) 109:1406–10. 10.1016/j.jada.2009.05.011 19631047

[20] DiNicolantonio JJ Niazi AK Lavie CJ O'Keefe JH Ventura HO. Thiamine supplementation for the treatment of heart failure: a review of the literature. Congest Heart Fail. (2013) 19:214–22. 10.1111/chf.12037 23910704

[21] Tanaka T Kono T Terasaki F Kintaka T Sohmiya K Mishima T. Gene-environment interactions in wet beriberi: effects of thiamine depletion in CD36-defect rats. Am J Physiol Heart Circ Physiol. (2003) 285:H1546–553. 10.1152/ajpheart.00182.2003 12969879

